# Supplementary material for: Invasion Dynamics and Migration Patterns of Fall Armyworm (Spodoptera frugiperda) in Shaanxi, China
Source: Insects. 2025 Jun 11;16(6):620. doi: 10.3390/insects16060620 (PMC12194478; doi:10.3390/insects16060620)
Supplement: Supplementary file 1 [file insects-16-00620-s001.zip › insects-3647444-supplementary.pdf]

Supplement tables

Table S1 The first occurrence of FAW in different sites of Shaanxi in 2019

City	Occurrence County	Initial date	Occurrence area (mu)	Average rate of damaged plants (%)	Average larva number per 100 plants	larval stage (instar)	Estimated immigration date
Ankang	Xunyang (109°26'E, 32°52'N)	June 10th	1.2	0.3	1	2-4	May 29th
Ankang	Zhenping (109°26'E, 31°51'N)	June 27th	0.3	2.2	3	2-4	June 16th
Ankang	Hanyin (108°36'E, 32°50'N)	June 24th	0.3	1.1	2	3-4	June 15th
Shangluo	Luonan (110°21'E, 34°01'N)	July 4th	2	0.1	1	2-4	June 20th
Shangluo	An County (109°18'E, 33°18'N)	June 13th	20	3.1	6	2-4	May 30th
Shangluo	Shanyang (110°16'E, 33°27'N)	June 14th	1.4	3.1	4	2-4	June 4th
Hanzhong	Yang County (107°44'E, 33°07'N)	May 31th	0.4	7.1	3	2-4	May 21th
Hanzhong	Hantai District (106°55'E, 33°09'N)	June 4th	2.0	4.6	4	1-5	May 18th
Baoji	Mei County (107°50'E, 34°17'N)	July 1st	5.8	2.1	2	3-6	June 13th
Baoji	Fufeng (107°57'E, 34°17'N)	July 2nd	4.5	2.2	4	3-4	June 19th
Baoji	Feng County (106°32'E, 33°55'N)	July 15th	1.2	7.1	7	3-5	June 30th
Xianyang	Xingping (108°22'E, 34°14'N)	June 25th	1.1	0.1	1	2-3	June 15th
Xianyang	Jingyang (108°22'E, 34°14'N)	July 12th	1.7	0.4	1	2-4	June 30th
Weinan	Linwei (109°42'E, 34°36'N)	July 26th	1.3	0.1	1	2-6	June 28th
Weinan	Chencheng (109°57'E, 35°21'N)	June 23th	10	0.3	1	1-3	June 10th
Weinan	Pucheng (109°42'E, 34°48'N)	August 1st	12	0.5	1	2-4	July 10th

Table S2 The first occurrence of FAW in different sites of Shaanxi in 2020

City	Occurrence County	Initial date	Occurrence area (mu)	Average rate of damaged plants (%)	Average larva number per 100 plants	larval stage (instar)	Estimated immigration date
Ankang	Xunyang (109°32'E, 33°06'N)	July 3rd	2.9	0.8	2	3-4	June 17th
Ankang	Zhenping (109°26'E, 31°51'N)	June 28th	40	7	15	1-4	June 15th
Ankang	Hanyin (108°36'E, 32°50'N)	July 2nd	1.2	0.9	1	4-5	June 16th
Shangluo	Luonan (110°21'E, 34°01'N)	August 4th	95	2.1	1	4-5	July 18th
Shangluo	An County (109°18'E, 33°18'N)	July 3rd	0.2	4.5	9	2-3	June 20th
Shangluo	Shanyang (110°16'E, 33°27'N)	July 4th	8	1	2	3-4	June 22th
Hanzhong	Mian County (106°27'E, 33°06'N)	July 2nd	7	2.5	4	3-4	June 20th
Hanzhong	Lueyang (106°26'E, 33°12'N)	July 6th	3	2.8	2	3-5	June 26th
Hanzhong	Ningqiang (106°03'E, 33°57'N)	June 30th	6.5	2.7	3	3-5	June 15th
Baoji	Mei County (107°50'E, 34°17'N)	July 20th	17.8	2	2	3-5	July 2nd
Baoji	Fufeng (107°57'E, 34°17'N)	July 21th	10.5	2.4	1	2-3	July 10th
Baoji	Feng County (106°32'E, 33°55'N)	July 5th	17	3.2	1	2-3	June 25th
Xianyang	Xingping (108°22'E, 34°14'N)	August 8th	2	0.2	1	3-5	July 15th
Xianyang	Jingyang (108°22'E, 34°14'N)	July 29th	4	1.1	1	4-5	July 13th
Weinan	Linwei (109°42'E, 34°36'N)	August 8th	66	0.1	1	2-3	August 1st
Weinan	Chencheng (109°57'E, 35°21'N)	July 27th	15	1.2	1	1-3	July 16th
Weinan	Pucheng (109°42'E, 34°48'N)	July 6th	13	0.5	1	2-4	June 22th

Table S3 The first occurrence of FAW in different sites of Shaanxi in 2021

City	Occurrence County	Initial date	Occurrence area (mu)	Average rate of damaged plants (%)	Average larva number per 100 plants	larval stage (instar)	Estimated immigration date
Ankang	Xunyang (109°32'E, 33°06'N)	July 5th	5.2	1.5	3	2-4	June 24th
Ankang	Zhenping (109°26'E, 31°51'N)	July 9th	4	2	2	1-2	June 29th
Ankang	Hanyin (108°36'E, 32°50'N)	July 8th	3.8	1.2	2	1-2	June 28th
Shangluo	Luonan (110°21'E, 34°01'N)	July 20th	82	2.5	4	2-4	July 5th
Shangluo	An County (109°18'E, 33°18'N)	July 14th	1.5	3	5	2-4	July 2nd
Shangluo	Shanyang (110°16'E, 33°27'N)	July 6th	26	1.4	4	3-6	June 16th
Hanzhong	Zhenba (107°44'E, 32°27'N)	July 8th	3.8	1.5	5	2-4	June 26th
Hanzhong	Ningqiang (106°03'E, 33°57'N)	July 6th	7.2	3.5	3	2-4	June 25th
Hanzhong	Chenggu (107°11'E, 33°27'N)	June 22th	7	2.6	4	3-5	June 8th
Baoji	Mei County (107°50'E, 34°17'N)	July 7th	137	2.3	3	3-4	June 22th
Baoji	Fufeng (107°57'E, 34°17'N)	August 8th	35	1.2	1	4-5	July 25th
Baoji	Feng County (106°32'E, 33°55'N)	July 19th	4.9	1	2	1-5	June 28th
Xianyang	Xingping (108°22'E, 34°14'N)	July 6th	2	0.6	1	3-4	June 22th
Xianyang	Jingyang (108°22'E, 34°14'N)	August 9th	4	0.6	1	4-5	July 21th
Weinan	Linwei (109°42'E, 34°36'N)	August 3rd	21	1.3	1	1-2	July 25th
Weinan	Pucheng (109°42'E, 34°48'N)	August 10th	56	0.3	1	2-4	July 27th

Table S4 The first occurrence of FAW in different sites of Shaanxi in 2022

City	Occurrence County	Initial date	Occurrence area (mu)	Average rate of damaged plants (%)	Average larva number per 100 plants	larval stage (instar)	Estimated immigration date
Ankang	Xunyang (109°32'E, 33°06'N)	July 21th	3.2	0.2	1	3-4	July 9th
Ankang	Zhenping (109°26'E, 31°51'N)	July 13th	23	3	3	2-4	June 30th
Ankang	Hanyin (108°36'E, 32°50'N)	July 26th	7.6	1.3	1	4-5	July 12th
Shangluo	Luonan (110°21'E, 34°01'N)	August 2nd	6.3	2.3	3	2-3	July 25th
Shangluo	Zhen’an (109°10'E, 33°31'N)	July 26th	20	2.3	4	2-3	July 15th
Shangluo	Shanyang (110°16'E, 33°27'N)	July 13th	130	0.4	1	3-4	July 1st
Hanzhong	Mian County (106°54'E, 33°09'N)	July 14th	5	7	3	3-4	June 30th
Hanzhong	Ningqiang (106°03'E, 33°57'N)	July 11th	3.7	2.3	2	2-4	June 28th
Baoji	Mei County (107°50'E, 34°17'N)	August 15th	63.9	0.5	1	2-5	July 25th
Xianyang	Xingping (108°22'E, 34°14'N)	July 29th	2	0.1	1	2-3	July 14th
Xianyang	Jingyang (108°22'E, 34°14'N)	August 3rd	16.8	0.1	1	3-4	July 22th
Weinan	Linwei (109°42'E, 34°36'N)	August 4th	2.2	0.1	1	1-2	July 26th

Table S5 The first occurrence of FAW in different sites of Shaanxi in 2023

City	Occurrence County	Initial date	Occurrence area (mu)	Average rate of damaged plants (%)	Average larva number per 100 plants	larval stage (instar)	Estimated immigration date
Ankang	Xunyang (109°32'E, 33°06'N)	July 20th	8.5	0.2	1	3-4	July 8th
Ankang	Zhenping (109°26'E, 31°51'N)	June 26th	80	3	4	2-4	June 14th
Ankang	Hanyin (108°36'E, 32°50'N)	July 24th	6.5	1.8	2	3-4	July 12th
Shangluo	Luonan (110°21'E, 34°01'N)	July 20th	260	0.3	1	1-4	July 7th
Shangluo	An County (109°18'E, 33°18'N)	July 21th	6	1.3	2	3-4	July 9th
Shangluo	Shanyang (110°16'E, 33°27'N)	July 18th	20	0.3	1	2-4	July 6th
Hanzhong	Mian County (106°54'E, 33°09'N)	June 20th	1.2	1.1	2	2-5	June 1st
Hanzhong	Yang County (107°44'E, 33°07'N)	June 26th	0.3	5.1	6	3-5	June 16th
Hanzhong	Ningqiang (106°03'E, 33°57'N)	July 6th	1.1	0.1	1	4-5	June 20th
Baoji	Feng County (106°32'E, 33°55'N)	July 27th	3.6	0.3	1	3-5	July 2nd
Xianyang	Xingping (108°22'E, 34°14'N)	July 31th	101	1.6	1	4-5	July 2nd
Xianyang	Jingyang (108°22'E, 34°14'N)	August 2nd	320	0.1	1	4-5	July 10th

Supplementary Material Results: FAW Migration Trajectory Simulation

1. 2019 FAW Migration Trajectory Simulation in Shaanxi

The backward trajectory simulation for the first and second nights of FAW invasion into Hantai District, Hanzhong City in 2019 showed that the drop points were distributed in northeastern Sichuan (Fig.S1A). The invasion time of FAW into Yang County, Hanzhong was similar to that of Hantai District, with the first night drop points distributed at the border of Sichuan and Chongqing, and the second night drop points distributed at the border of Hubei, Hunan and Chongqing (Fig.S1B).

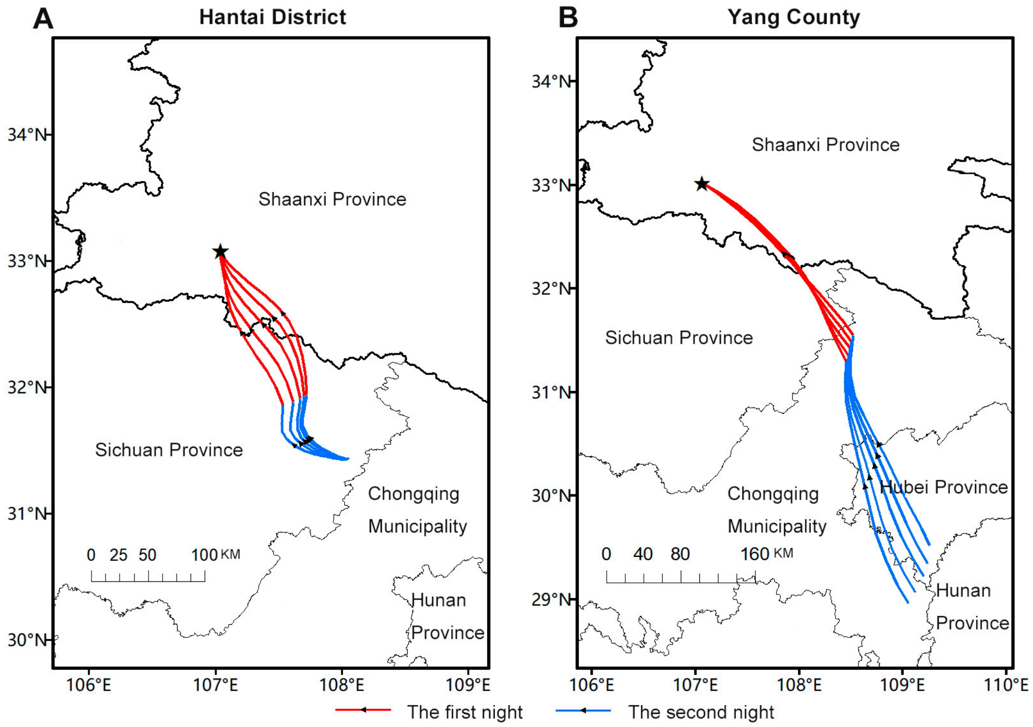

Figure S1 Backward trajectories of FAW in different Areas of Hanzhong City

The backward trajectory simulation analysis for FAW invasion into Ankang City showed that the drop points for the first and second nights of FAW invasion into Xunyang County in 2019 were distributed in northeastern Sichuan (Fig.S2A). The invasion time of FAW into Zhenping County was in mid-June, with the first night drop points in Chongqing and the second night drop points distributed at the border of Chongqing and Hubei (Fig.S2B).

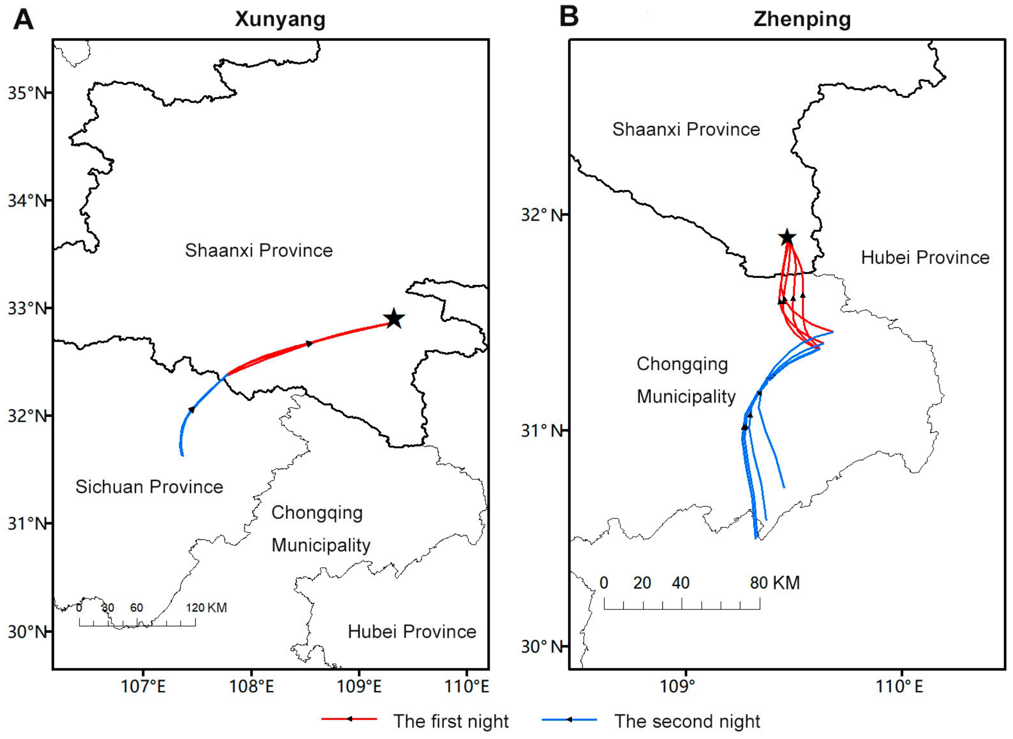

Figure S2 Backward trajectories of FAW in different Areas of Ankang City

The first night drop points for FAW invasion into Zhen'an County, Shangluo City were in northeastern Sichuan, and the second night drop points were at the border of Chongqing and Sichuan (Fig.S3A). The first night drop points for FAW invasion into Luonan County were in central Henan, and the second night drop points were at the border of Anhui and Henan (Fig.S3B).

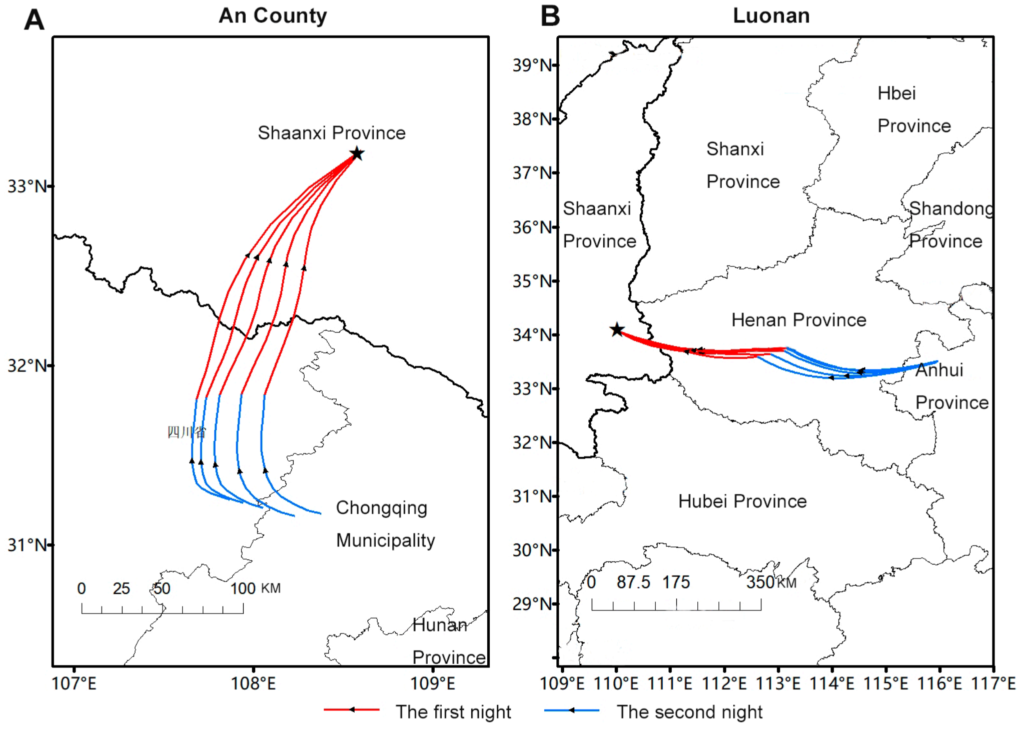

Figure S3 Backward trajectories of FAW in different Areas of Shangluo City

As 2019 was the first year of FAW invasion in China and Shaanxi, the backward trajectory simulation for FAW invasion into Guanzhong area was conducted. The results showed that the FAW might have invaded the Guanzhong area in late June. The drop points for the first and second nights of FAW invasion into Xingping City were at the border of Shaanxi, Henan and Hubei (Fig.S4A). The first night drop points for FAW invasion into Chengcheng County, Weinan City were mainly in central Henan, and the second night drop points were also within Henan Province (Fig.S4B). In June 2019, FAW invasions were also reported in Henan, which could provide a source of infestation. Based on the simulation results, the first batch of FAW invading the Guanzhong area in 2019 might have originated from Henan and Hubei.

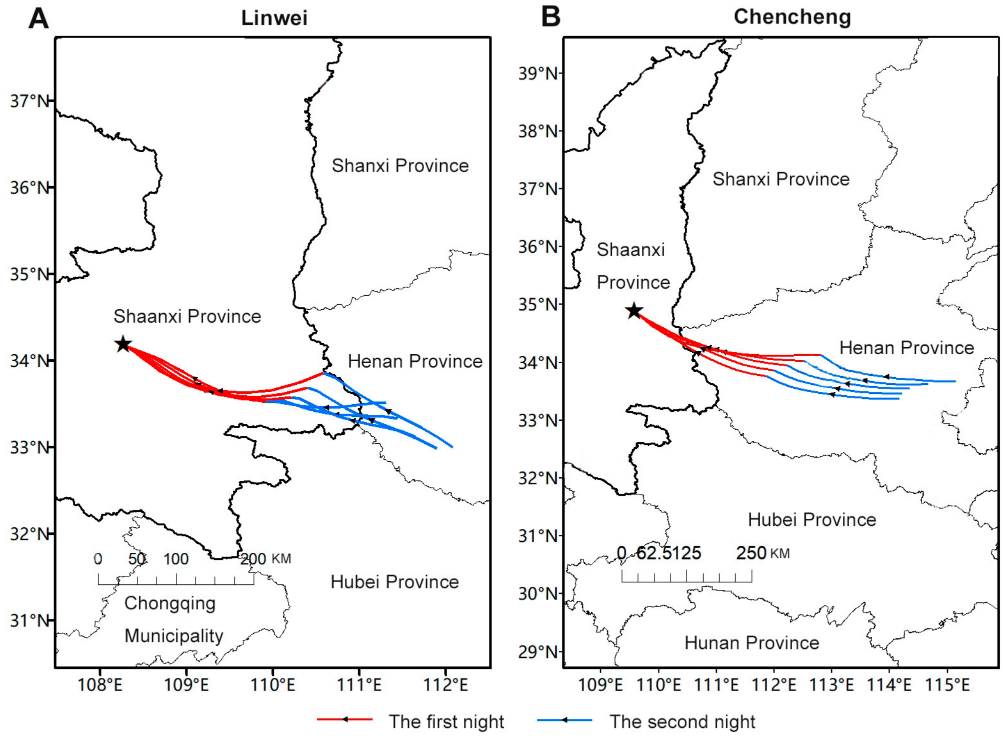

Figure S4　Backward trajectories of FAW in different Areas of Weinan City

2. 2020 FAW Migration Trajectory Simulation in Shaanxi

The FAW invasion period in Hanzhong in 2020 was also estimated to be in mid-June. Due to heavy rainfall on June 16, it was speculated that it might have affected FAW invasion. Therefore, the simulation date for backward trajectory was adjusted to June 14-15. The simulation showed that the drop points for the first night of FAW invasion into Ningqiang County, Hanzhong were in northeastern Sichuan, and the drop points for the second night were in Chongqing and Guizhou (Fig.S5A). If the FAW had migrated for two or even three consecutive nights, the first invasion of FAW into Hanzhong in 2020 might have originated from Sichuan or might have migrated across provinces from Guizhou. The drop points for the first and second nights of FAW invasion into Lueyang County were both in Sichuan (Fig.S5B).

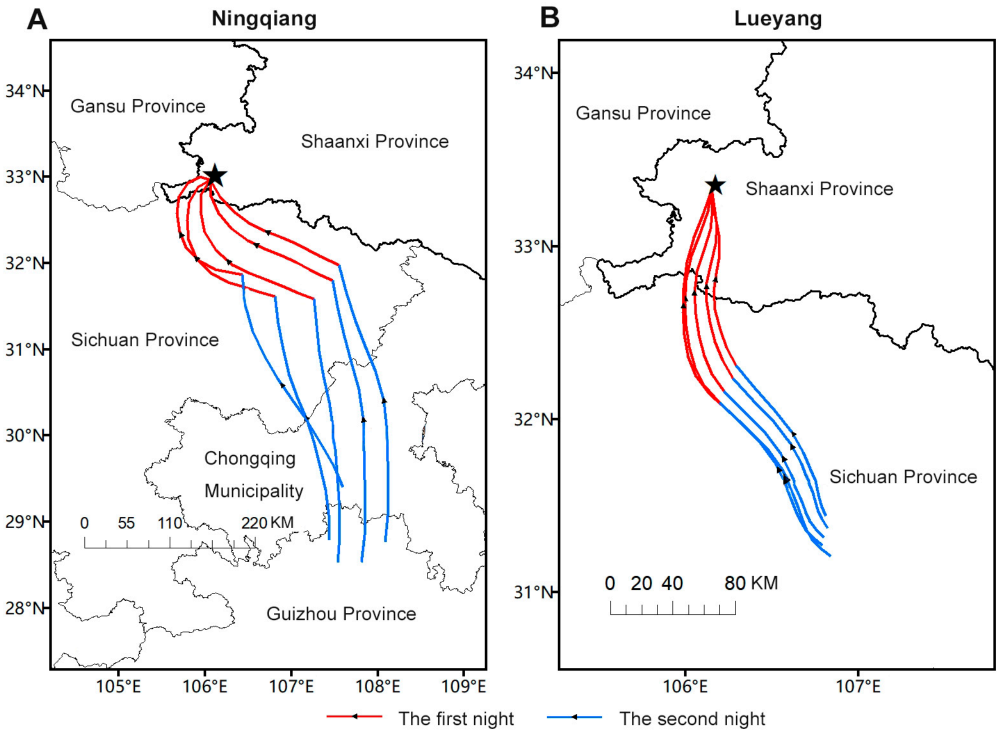

Figure S5 Backward trajectories of FAW in different Areas of Hangzhong City

The first batch of FAW invasion into Ankang City in 2020 was estimated to be in mid-June. However, due to heavy rainfall in Ankang on June 17, the backward trajectory simulation for FAW was adjusted to before June 17. The drop points for the first and second nights of FAW invasion into Zhenping County, Ankang were in Hubei and Chongqing (Fig.S6A). The drop points for the first night of FAW invasion into Xunyang County were in Hubei, and the drop points for the second night were in Hunan (Fig.S6B). During the invasion window period, there were significant changes in wind direction at night, leading to considerable differences in the direction of backward trajectories on different dates. This greatly affected the accuracy of backward trajectory simulation and the identification of FAW source areas.

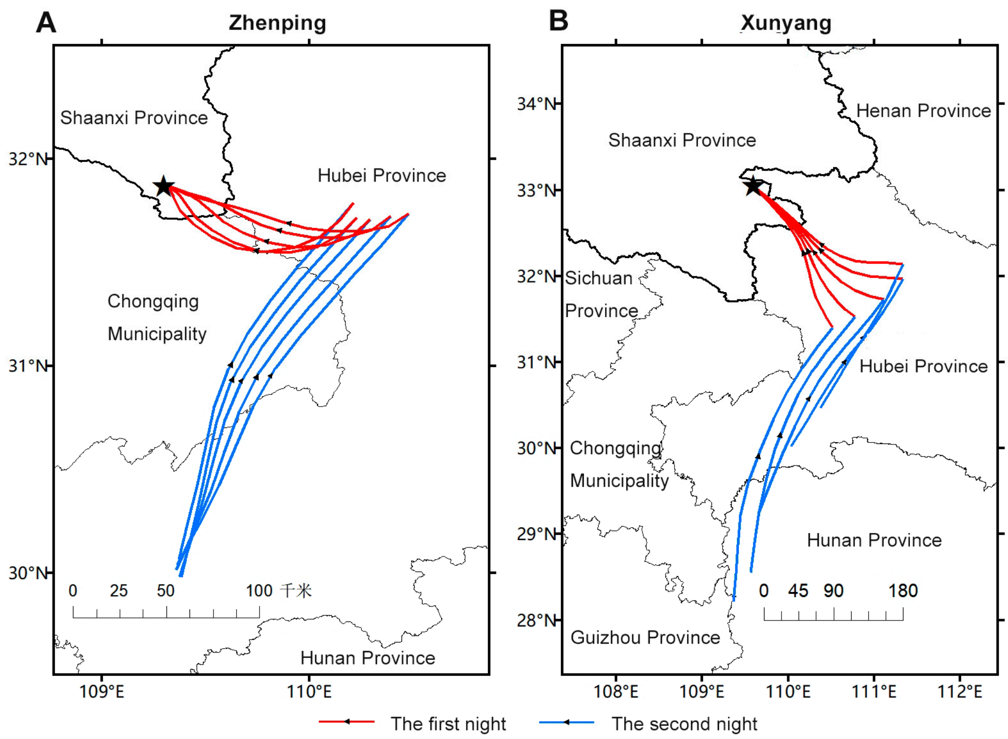

Figure S6 Backward trajectories of FAW in different Areas of Ankang City

Backward trajectory simulation was conducted for FAW populations invading Shangluo in 2020. The results showed that the drop points for the first night of FAW invasion into An County were at the border of Shaanxi and Hubei, and the drop points for the second night were in Hubei (Fig.S7A). The FAW invasion into Luonan County was nearly one month apart from the other two regions. The drop points for the first and second nights of FAW invasion into Luonan County were in southern Henan (Fig.S7C), and the direction of the backward trajectory was similar to that of Shang County (Fig.S7B). The first FAW in Luonan County was found one month later than in the other two regions. Based on the simulation results, it was speculated that the FAW population in Luonan County might have originated from the southern mountainous areas of Henan. On the day of FAW invasion into Shangluo City, there was light rainfall in various regions of Shangluo, which might have affected FAW landing.

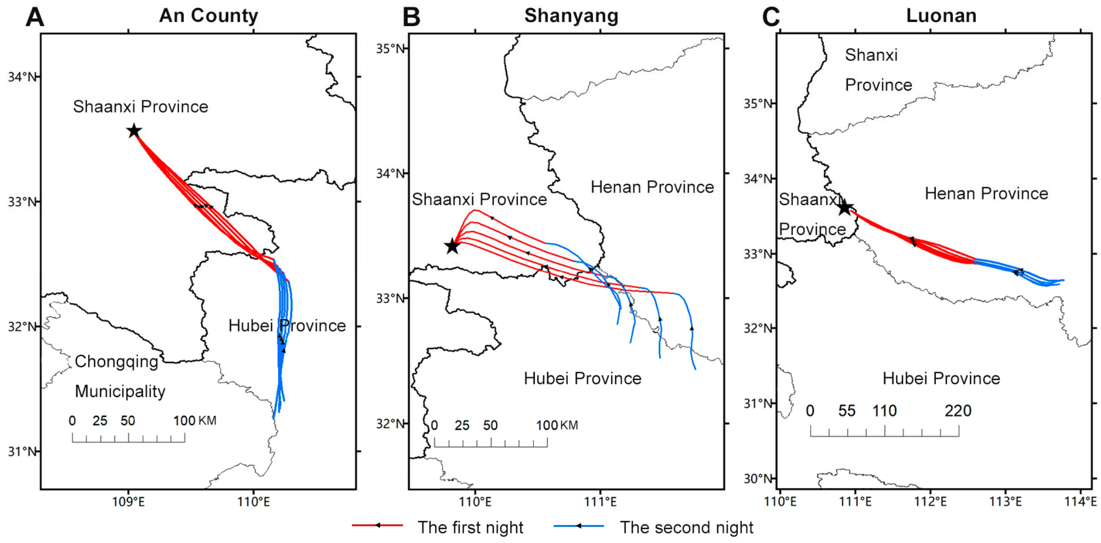

Figure S7 Backward trajectories of FAW in different Areas of Shangluo City

3. 2021 FAW Migration Trajectory Simulation in Shaanxi

In 2021, the first FAW in Hanzhong City was detected earlier than in 2020, with the estimated first invasion date in early June. Based on FAW infestation data, Chenggu County was the earliest to report FAW damage. The drop points for the first night of FAW invasion into Chenggu County were in northern Sichuan, and the drop points for the second night were at the border of Sichuan and Chongqing (Fig.S8A). Due to heavy rainfall in Hanzhong in mid-June, the FAW invasion into Ningqiang and Zhenba Counties was delayed until late June. The drop points for the first night of FAW invasion into Zhenba County were at the border of Sichuan and Chongqing, and the drop points for the second night were in Hubei (Fig.S8B). According to the simulation results, the FAW population in Zhenba County might have originated from Hubei and migrated across Chongqing and Sichuan over two consecutive nights (Fig.S8C).

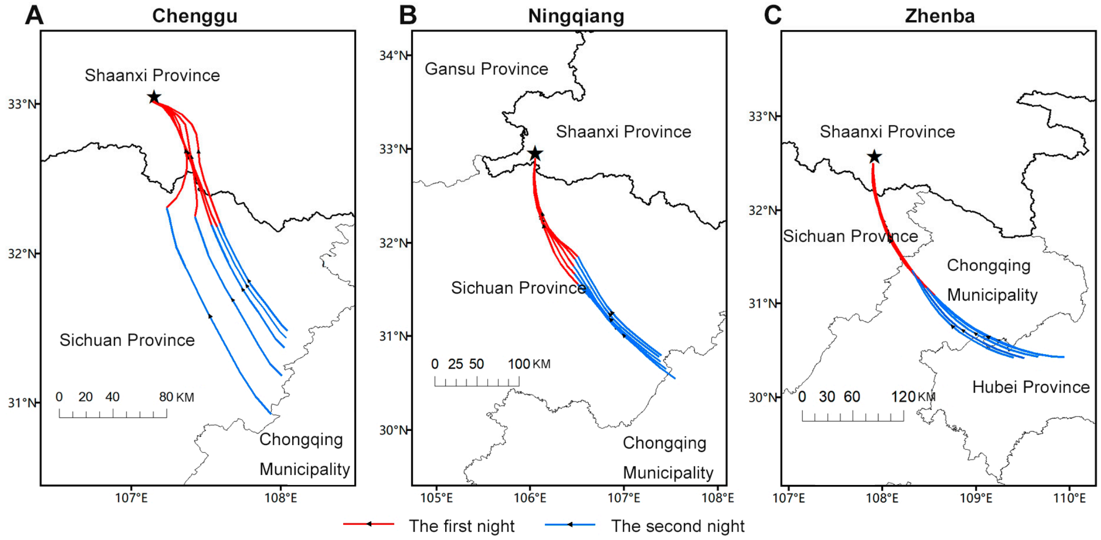

Figure S8 Backward trajectories of FAW in different Areas of Hangzhong City

In late June 2021, FAW invaded Ankang City. However, due to heavy rainfall on June 25 and

26, the simulation time for FAW backward trajectory was adjusted. The drop points for the first night of FAW invasion into Xunyang County were in Hubei, and it was also possible that the FAW originated from Henan and migrated for two consecutive nights (Fig.S9A). The drop points for the first and second nights of FAW invasion into Zhenping County were both in Hubei (Fig.S9B).

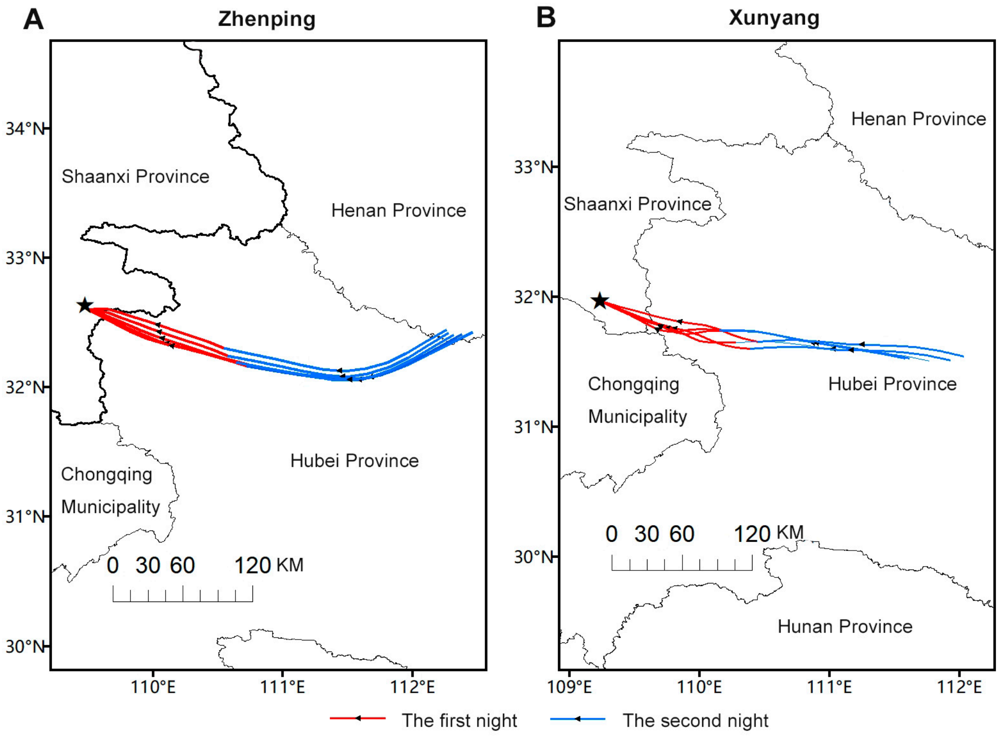

Figure S9 Backward trajectories of FAW in different Areas of Ankang City

According to FAW infestation data, similar to the situation in Shangluo City in 2021, the duration of FAW invasion into Shangluo was relatively long, with the first FAW damage detected in different districts and counties spanning a large time frame, from mid-June to mid-July. During this period, there was not much rainfall in Shangluo. Based on the simulation analysis of backward trajectories for FAW invasion into different regions of Shangluo in 2021, despite the large time span, the direction of the FAW backward trajectories did not change significantly. The drop points for the first night of FAW invasion into An County were at the border of Hubei and Henan, and the drop points for the second night were in Henan (Fig.S10A). The simulation results for Shanyang County were similar to those for An County (Fig.S10B). The drop points for the first and second nights of FAW invasion into Luonan County were both in Henan (Fig.S10C).

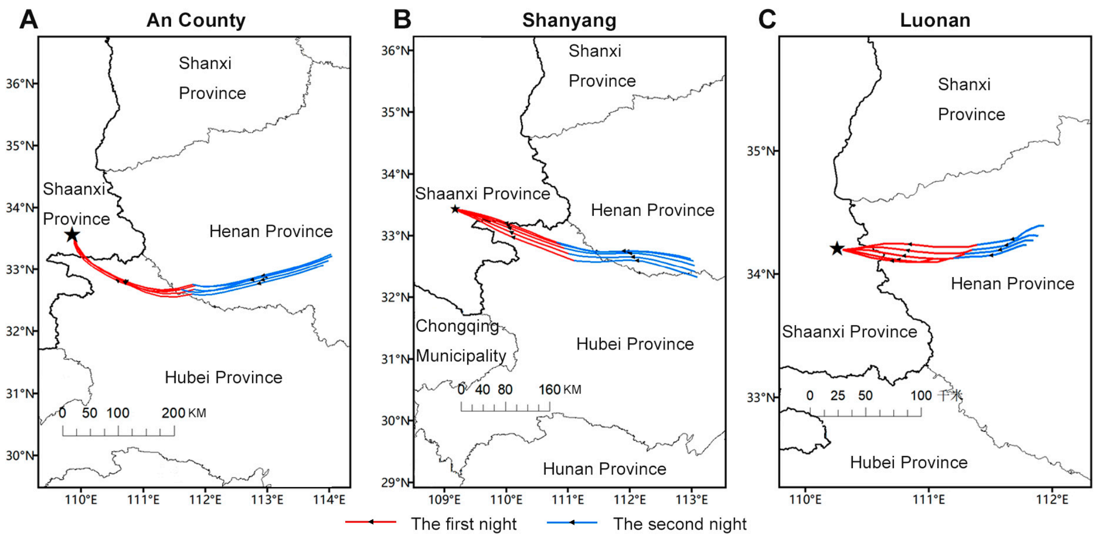

Figure S10　Backward trajectories of FAW in different Areas of Shangluo City

4. 2022 FAW Migration Trajectory Simulation in Shaanxi

In 2022, the first FAW in Shaanxi was detected later than in previous years. It was estimated that FAW began to migrate into Hanzhong and Ankang in late June. Combining precipitation data, the backward trajectory simulation for FAW invasion into Hanzhong City showed that the drop points for the first and second nights of FAW invasion into Ningqiang County were in Sichuan (Fig.S11A), and the drop points for the first night of FAW invasion into Zhenba County were in Sichuan, with the drop points for the second night at the border of Sichuan and Chongqing (Fig.S11B).

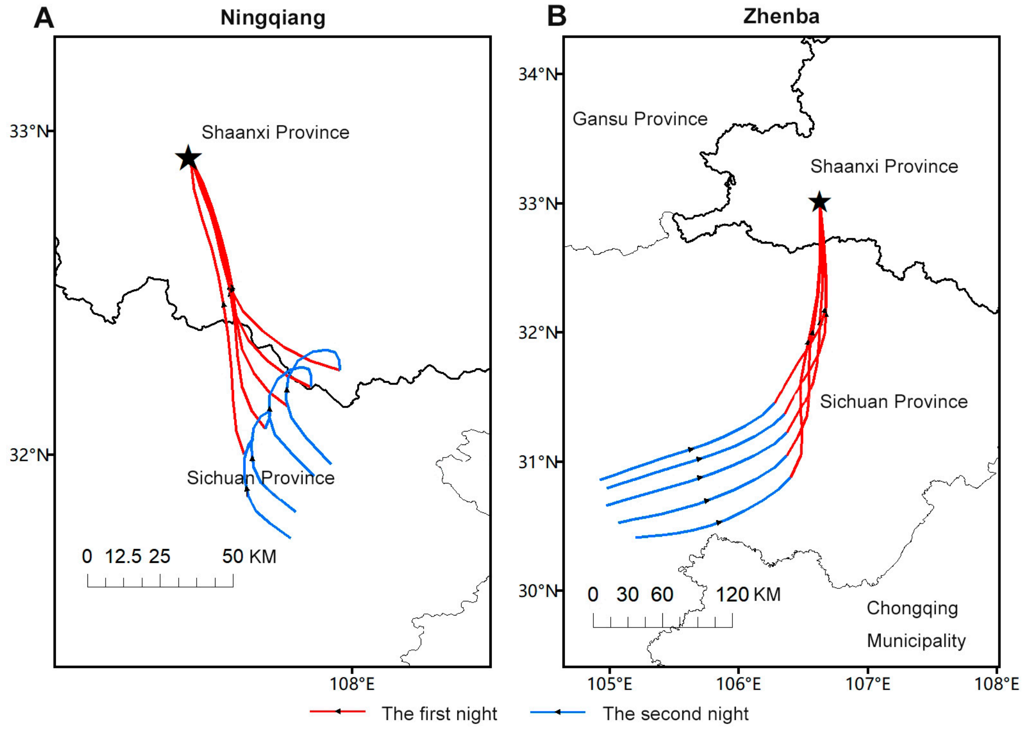

Figure S11　Backward trajectories of FAW in different Areas of Hanzhong City

The first FAW in Ankang City was detected on July 13, 2022. Based on infestation data, the

estimated migration date was late June. Combining precipitation data in Ankang City (Fig.S12), the simulation results showed that the drop points for the first and second nights of FAW invasion into Zhenping County were within Shaanxi Province(Fig.S12A), suggesting a possible local origin. The drop points for the first night of FAW invasion into Xunyang County were in Hubei, and the drop points for the second night were at the border of Henan and Hubei (Fig.S12B). The drop points for the first night of FAW invasion into Han County were in Chongqing, and the drop points for the second night were in Guizhou (Fig.S12C), indicating that FAW might have originated from Guizhou and migrated across Chongqing.

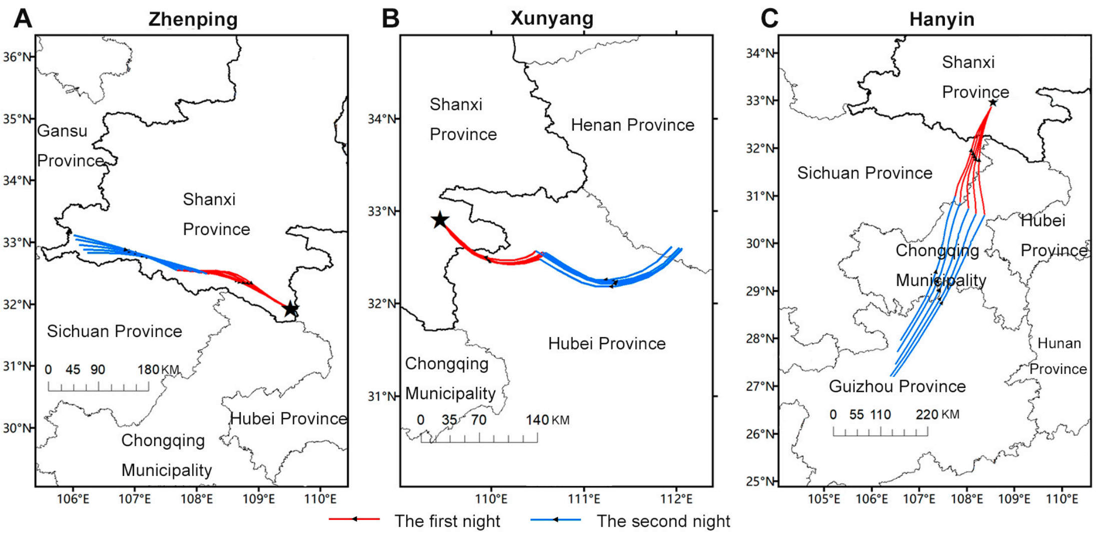

Figure S12 Backward trajectories of FAW in different Areas of Ankang City

According to FAW infestation data, the duration of FAW invasion into Shangluo in 2022 was relatively long, with the first FAW damage detected in different districts and counties spanning nearly one month, from late June in Shanyang County to late July in Luonan County. During the FAW migration period, there was not much rainfall in Shangluo (Fig.S13). The simulation results of backward trajectories for FAW invasion into different regions of Shangluo in 2022 indicated that the drop points for the first night of FAW invasion into Shanyang County were in Hubei, and the drop points for the second night were at the border of Hubei and Chongqing (Fig.S13A). The simulation results for FAW invasion into Luonan County were consistent with the western migration route of FAW in China, with the population originating from Sichuan and Chongqing (Fig.S13B).

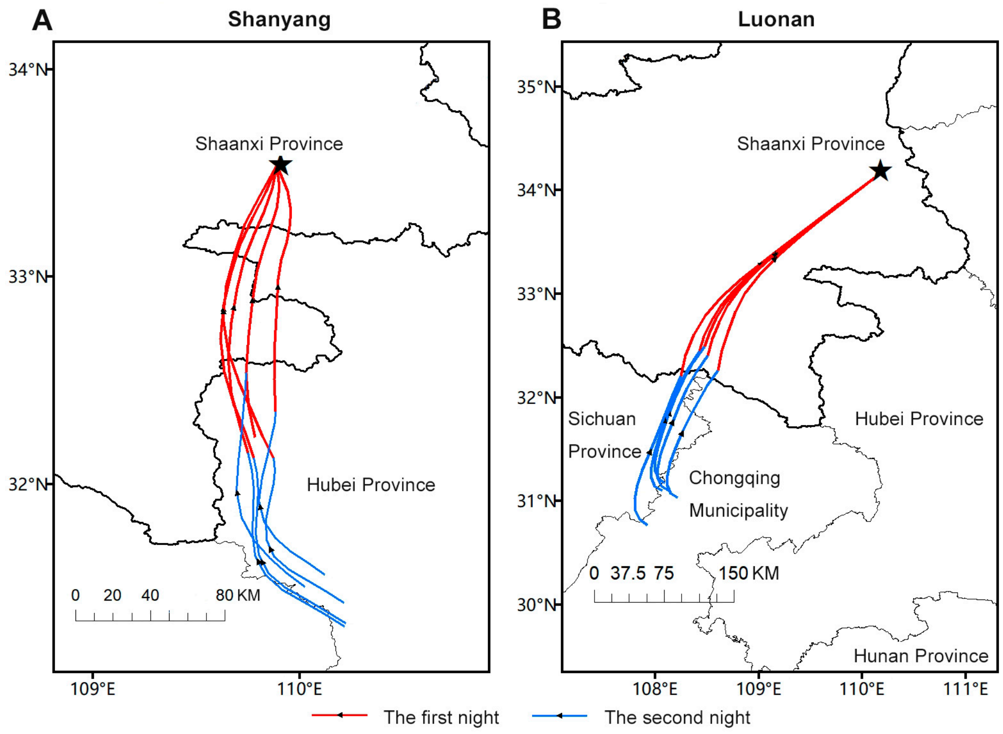

Figure S13 Backward trajectories of FAW in different Areas of Shangluo City

5. 2023 FAW Migration Trajectory Simulation in Shaanxi

In 2023, based on infestation data, FAW was estimated to have first migrated into Hanzhong City in early June. However, during the simulation of backward trajectories for FAW invasion into Mianning County, Hanzhong, it was found that no effective simulation trajectories could be obtained for the estimated migration period. Therefore, the simulation time was advanced. The drop points for the first night of FAW invasion into Mianning County were in Sichuan, and the drop points for the second night were at the border of Hubei and Chongqing (Fig.S14A). The drop points for the first night of FAW invasion into Yang County were in Sichuan, and the drop points for the second night were in Chongqing (Fig.S14B).

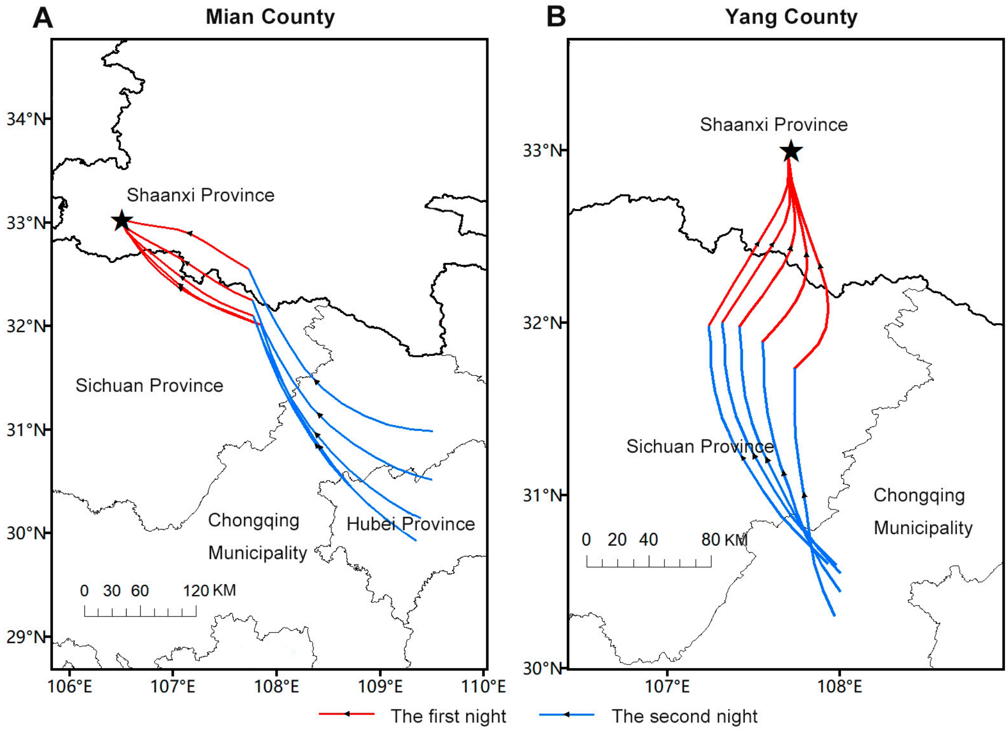

Figure S14 Backward trajectories of FAW in different Areas of Hanzhong City

According to the simulation results, the origin of FAW invading Ankang City in 2023 was Sichuan and Chongqing (Fig.S15A). Due to the strong migration ability of FAW, although the direct origin of FAW in Ankang City was Sichuan and Chongqing, it is also possible that FAW populations from Guizhou migrated northward via Chongqing and Hubei at night with the seasonal winds (Fig.S15B).

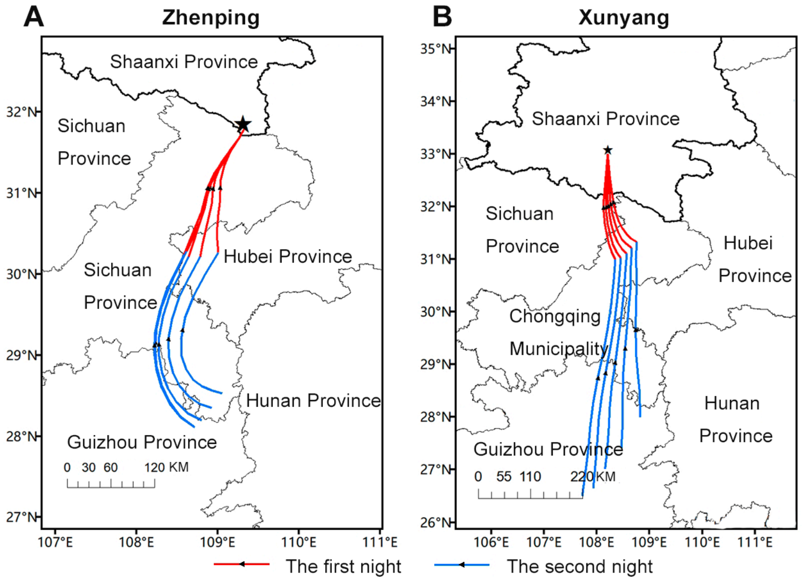

Figure S15 Backward trajectories of FAW in different Areas of Ankang City
